# Natural Killer Cell-Derived Extracellular Vesicles: Novel Players in Cancer Immunotherapy

**DOI:** 10.3389/fimmu.2021.658698

**Published:** 2021-05-21

**Authors:** Feifeng Wu, Min Xie, Marady Hun, Zhou She, Cuifang Li, Senlin Luo, Xiaoyu Chen, Wuqing Wan, Chuan Wen, Jidong Tian

**Affiliations:** Department of Pediatrics, The Second Xiangya Hospital, Central South University, Changsha, China

**Keywords:** natural killer cell, cancer, extracellular vesicle, immunotherapy, cell-free therapy

## Abstract

Natural killer (NK) cells are critical components of host innate immunity and function as the first line of defense against tumors and viral infection. There is increasing evidence that extracellular vesicles (EVs) are involved in the antitumor activity of NK cells. NK cell-derived EVs (NKEVs) carrying cargo such as cytotoxic proteins, microRNAs, and cytokines employ multiple mechanisms to kill tumor cells, but also exhibit immunomodulatory activity by stimulating other immune cells. Several studies have reported that NKEVs can reverse immune suppression under tolerogenic conditions and contribute to NK-mediated immune surveillance against tumors. Thus, NKEVs are a promising tool for cancer immunotherapy. In this review, we describe the biological effects and potential applications of NKEVs in antitumor immunity.

## Introduction

Extracellular vesicles (EVs) are heterogeneous, membrane-bound phospholipid vesicles that are actively released by most cell types including immune and cancer cells (1). EVs can be classified based on their biogenesis, size, and biophysical properties. Unless otherwise specified, in this review EVs refer to exosomes and microvesicles (MVs). As mediators of intercellular communication, EVs carry specific bioactive agents that alter gene expression in recipient cells.

Natural killer (NK) cells belong to the innate lymphoid cell family and are involved in early defense against tumors and infection, which does not require priming. In human peripheral blood, bone marrow, and tissues, NK cells can be identified by the absence of surface expression of T cell receptor (TCR) and associated cluster of differentiation (CD)3 molecules, and by the expression of neural cell adhesion molecule (also known as CD56) (2). NK cells are divided into two subgroups with functional and phenotypic properties: CD56^dim^ cells are found in the circulation and are mainly cytotoxic, whereas CD56^bright^ cells dominate in secondary lymphoid tissues and play an immunomodulatory role by producing high levels of cytokines (3). NK cell function is controlled by activating receptors (e.g., natural cytotoxicity receptors [NCRs], natural killer group [NKG]2D, DNAX accessory molecule [DNAM]1, and fragment crystallizable gamma receptor [FcγRIIIA]/CD16A) and inhibitory receptors. In general, activation of individual receptors cannot induce cytotoxicity by NK cells except in the case of CD16A, which binds to the Fc region of antibody-coated cells and elicits antibody-dependent cell-mediated cytotoxicity. Killer cell immunoglobulin-like receptors (KIRs), which are inhibitory receptors for major histocompatibility complex (MHC) class I molecules in humans, provide signals for the tolerance of healthy cells by NK cells and contribute to the licensing process—i.e., MHC-dependent NK cell education (4). Besides their direct cytotoxic effect, NK cells produce cytokines and chemokines such as interferon (IFN)-γ, tumor necrosis factor (TNF), interleukin (IL)-6, granulocyte-macrophage colony-stimulating factor (GM-CSF), and C-C motif chemokine ligand (CCL)5 that provide an important link between innate and adaptive immunity (5).

There is increasing evidence that immune cells including NK cells release EVs into the extracellular space to modulate tumor immunity. NKEVs have also been detected in serum. This suggests that the functions of NK cells go beyond the traditional cell–cell interactions and paracrine signaling (6). In addition to their antitumor effects, NKEVs have immunomodulatory properties (7) and likely play a role in cancer immunotherapy. In this review, we describe the biological effects and potential applications of NKEVs in tumor immunity.

## Isolation and Characterization of NKEVs

### Cellular Source of NKEVs

NKEVs are exosomes or MVs that are released by NK cells and can be isolated from the cell culture supernatant—including that of resting NK cells (6), ex vivo-expanded NK cells (6–13), NK92 cells (8–11), and NK cell-enriched lymphocytes (12)—or from human plasma (6, 7). Peripheral blood mononuclear cells (PBMCs) and NK92 cells are the two major cellular sources of NKEVs. EVs derived from NK92 cells were shown to express lower levels of cytotoxic proteins than ex vivo-expanded PBMCs in several studies, but there is no evidence for the superiority of the latter cells. Resting NK cells constitutively release exosomes with no significant differences in the amounts or in surface marker expression relative to activated NK cells (6). Recent studies have favored using activated NK cells as a source of EVs, possibly because of the large numbers that can be obtained by *in vitro* expansion. Human serum is not an ideal source of NKEVs because of the low number of circulating NK cells and exosomes. However, several studies have proposed isolating NKEVs from human sera. Consistent with the proportion of NK cells among PBMCs, the level of circulating tumor susceptibility gene (TSG)101^+^CD56^+^ NKEVs was lower in melanoma patients than in healthy donors, suggesting that they reflect changes in NK cell profiles (6, 7).

### Isolation and Definition of NKEVs

There is currently no standard method for isolating exosomes from NK cell cultures or plasma (**Table 1**). Although ultracentrifugation is the most commonly used technique, some commercial kits such as the ExoQuick and exoEasy Maxi kits are also available (16, 19). One group combined polyethylene glycol 800 precipitation and buoyant density gradient centrifugation for large-scale isolation of EVs from NK cells expanded in culture (50 ml to 5 l of conditioned medium) (16). Each method has advantages and limitations and can achieve different degrees of EV purity and concentration. The isolated vesicles must be identified by transmission electron microscopy, nanosight tracking analysis, and western blotting (20). There is no clear molecular definition of NKEVs. In addition to expressing typical exosome markers (e.g., CD63, Rab5b, and TSG101), most studies have used CD56 as a marker to identify NKEVs isolated from CD56^+^ NK cells. Additionally, receptors of NK cells such as natural killer cell P30-related protein (NKp30), NKp46, NKp44, NKG2D, and DNAM1 were shown to be expressed by NKEVs in some studies and may be useful for their identification (6, 12, 14, 17). However, definitive markers have yet to be established as CD56 is not exclusive to NK cells and a subset of NK cells are CD56^−^.

**Table 1:** Isolation, characterization, and biological effects of NKEVs.

Source	NK cell preparation	Method	Yield	Diameter, nm	Markers		Potential effects				Ref
					Exo-related	NK-related	Biomolecules	Cell type/model	Mechanisms	Effects	
**Human PBMCs**	CD3/CD4/CD8/CD20/CD14-depleted PBMCs cocultured with cobalt-irradiated RPMI8866 cells stimulated with IL-2	UC	5.36 ± 3 μg/1×10^6^ cells	40–100	MHC I; Rab5b; CD56	CD56; NKp30; NKp46; NKp44	mFasL; sFasL; perforin	Jurkat; K562; PHA-activated PBMCs	−	Dose- and time-dependent cytotoxicity	(6)
**NK92-MI cells**	−	UC	−	100–150	CD63; ALIX	−	Perforin; FasL; TNF-α	B16F10; melanoma xenograft mouse model	Activate extrinsic and intrinsic apoptotic pathways; inhibit MAPK signaling	Dose- and time- dependent cytotoxicity; inhibit proliferation; induce apoptosis; inhibit tumor growth and reduce tumor volume	(8)
**Human PBMCs**	Cobalt-irradiated B lymphoblastoid RPMI8866 cells, IL-2 stimulation	UC	2.1×10^4^ exo/cell; 0.6×10^3^ MV/cell	Exo: 124 ± 3.8; MV: 315.2 ± 4.8	TSG101	CD56	−	−	−	Activate PBMCs	(7)
**Human PBMCs**	Coculture with feeder cell line K562.mbIL21; IL-2/IL-15 stimulation	SEC	−	Mean 92.45	CD81; TSG101; HSP70	−	Perforin 1; granzyme A and B; miR-186	MYCN-amplified CHLA-136 and LAN-5; orthotopic NB mouse model	Downregulation of MYCN, AURKA, TGFBR1, and TGFBR2	Inhibit growth of neuroblastoma; counteract TGF-β–dependent immune escape	(13)
**Human PBMCs**	PHA, IL-2, or IL-15 stimulation	UC	0.62 ± 0.2 µg/1×10^6^ cellsx	135.9 ± 0.5	CD63; CD81; TSG101; CD16; CD69	CD16; NKp44; NKp30; NKp46; DNAM1	Perforin 1; granzyme A and B; IFN-γ; PD1	NALM-18	−	Rapid and efficient tumor cell killing	(14)
**NK92-MI cells**	−	UC	1.36 μg (1.86×10^6^ particles)/5×10^6^ cells	118 ± 33.1	CD63; ALIX	−	FasL; perforin	D54/F	Activate extrinsic and intrinsic apoptotic pathways; inhibit MAPK and PI3K signaling	Dose- and time-dependent cytotoxicity; inhibit tumor cell proliferation	(11)
**NK92-MI cells**	−	UC	1.58 μg (1.7×10^5^ particles)/10^7^ cells	106.9 ± 21.6	CD63; ALIX	−		U87-MG; glioblastoma xenograft mouse model	Activate extrinsic and intrinsic apoptotic pathways	Dose- and time-dependent cytotoxicity; inhibit tumor growth and reduce tumor volume	(15)
	IL-15 stimulation		4.22 μg (3.7×10^5^ particles)/10^7^ cells	118.2 ± 20.3							
**Human PBMCs**	CD3-depleted PBMCs cocultured with γ-irradiated K562 clone 9.mbIL21 cells; IL-2 stimulation	PEG8000+BDGC	20.6 ± 0.09×10^8^ particles/ml	155 ± 5.9	CD63; Rab5A;	−	Perforin; granulysin; granzyme A and B	SUP-B15; LA-255	Caspase-dependent apoptosis	Dose-dependent cytotoxicity	(16)
		ExoQuick	19.2 ± 2.09×10^8^ particles/ml	158 ± 1.7							
		UC	19.4 ± 0.05×10^8^ particles/ml	173 ± 13.6							
**Human PBMCs**	CD3^/^CD4-depleted PBMCs, IL-2/IL-12/IL-15/IL-21 stimulation, coculture with NB cells	UC	−	40–150	ALIX, TSG101	CD56; NKp30; NKG2D; NKp44; NKp46	−	SK-N-SH; CHLA-255	−	Enhance cytotoxicity of NK cells against neuroblastoma	(17)
**Human PBMCs**	Direct isolation	UC	−	60–150	TSG101; CD63	−	miR3607-3p	Mia PaCa-2; PANC-1	Possibly targets IL-26	Inhibit cell viability, proliferation, migration, and IL-26 production	(18)
**Mouse spleen**	Direct isolation as CD3^−^CD49b^+^ cells	exoEasy Maxi kit	−	50–150	CD63; CD81	−	miR-207	Astrocyte	miR-207/TLR4/ NF-κB signaling pathway	Inhibit proinflammatory cytokine release	(19)
**Human PBMCs**	NK-enriched lymphocytes	UC	−	−	CD40L; CD63; CD51; CD62	NKG2D; DNAM1; NKp44; NKp46	Fas; DR4; DR5; FasL; TRAIL; IFN-γ; TNF-α; IL-6	HepG2; SW-620; MKN-74; MCF-7; T98G; MCF-7–based breast cancer model	−	Dose-dependent cytotoxicity; suppress tumor growth *in vivo*	(12)
**NK92 cells**	IL-2 stimulation	UC	2.5 ± 0.3 μg/10^6^ cells	190–460	−	−	−	−	−	−	(10)
**NK92 cells**	−	UC	−	Mean 100	ALIX; TSG101; CD63	−	−	MCF-7	−	Inhibit tumor cell proliferation and migration; induce apoptosis	(9)

−, no available data; ALIX, ALG2-interacting protein X; ALL, acute lymphoblastic leukemia; BDGC, buoyant density gradient centrifugation; CD, cluster of differentiation; DNAM1, DNAX accessory molecule 1; DR, death receptor; exo, exosome; FasL, Fas ligand; HSP70, heat shock protein 70; IFN-γ, interferon gamma; IL, interleukin; MAPK, mitogen-activated protein kinase; mFasL, membrane-bound Fas ligand; MHC, major histocompabitility complex; miR, microRNA; MV, microvesicle; MYCN, MYCN proto-oncogene BHLH transcription factor; NB, neuroblastoma; NF-κB, nuclear factor kappa B; NK, natural killer; NKG2D, natural killer group 2D; NKp30/44/46, natural killer cell P30/44/46-related protein; PBMC, peripheral blood monocular cell; PD1, programmed death 1; PEG8000, polyethylene glycol 8000; PHA, phytohemagglutinin; PI3K, phosphatidylinositol 3-kinase; Ref, reference; RPMI, Roswell Park Memorial Institute; SEC, size exclusion chromatography; sFasL, soluble Fas ligand; TGF-β, transforming growth factor beta; TGFBR, transforming growth factor beta receptor; TLF4, Toll-like receptor 4; TNF-α, tumor necrosis factor alpha; TSG101, tumor susceptibility gene 101; TRAIL, TNF-related apoptosis-inducing ligand; UC, differential ultracentrifugation.

### Heterogeneity of NKEVs

The contents, size, and membrane composition of NKEVs are highly heterogeneous and dynamic depending on the cellular source, physiologic status, and environmental conditions. There have been few proteomic studies of NKEV contents, and most investigations have focused on several specific (mostly cytotoxic) proteins, which has masked the true heterogeneity of NKEVs. For example, EVs derived from *in vitro*-expanded NK cells have higher levels of cytotoxic proteins compared to those secreted by the NK92 cell line (21), whereas EVs derived from inactivated NK cells have lower levels of cytotoxic proteins compared to those isolated from activated NK cells (13). IL-15–treated NK cells produce more exosomes, which may be associated with the upregulation of Rab27a (15), a cytosolic protein that regulates different steps of vesicular trafficking including budding, motility, or docking of vesicular transport intermediates to the acceptor membrane (22). In contrast, transforming growth factor (TGF)-β–treated NK cells release vesicles containing lower levels of cytotoxic proteins. It was also reported that NK cells secrete exosomes irrespective of their activation status (6). Determining the composition and molecular profiles of NKEVs can provide more detailed insight into the extent of their heterogeneity.

Like NK cells, NKEVs exert time- and dose-dependent cytotoxicity toward hematologic tumor cells. However, findings from solid tumors have been more variable. For example, breast cancer cells (SKBR3) and melanoma cells (501mel) were shown to resist lysis of exosomes released by resting or activated NK cells even over a long period of coculture (6). However, it was later shown that exosomes derived from NK-92 MI cells had antitumor effects on melanoma both *in vitro* (B16F10 cells) and *in vivo* (8), and those isolated from IL-2/IL-15–activated NK cells showed cytotoxicity in breast cancer cell lines (MDA-MB-231/F and MCF-7) (12, 15, 16). The heterogeneous content of exosomes from different cellular sources may account for these inconsistent observations.

### Memory-Like NKEVs

NK cells have traditionally been considered as innate immune cells that function in an antigen-independent manner and do not develop immunologic memory, although the latter point has been challenged by recent evidence. For example, following murine cytomegalovirus (MCMV) infection, mouse NK cells acquired traits of adaptive immunity such as expansion of virus/m157-specific NK cell subsets and long-lasting secondary responses including increased protection against MCMV compared to naïve NK cells; this immunologic memory was generally hapten-specific (23). Priming with cytokines (e.g., IL15 or IL12) elicited NK cell antigen-nonspecific memory such as enhanced cytokine production and cytotoxicity (24, 25). NK cells may also produce memory-like NKEVs under some conditions, which usually requires a pathogen or proinflammatory cytokines. For example, exosomes released by IL-15–treated NK cells showed upregulation of TNF-related apoptosis-inducing ligand (TRAIL), Nkp46, and Nkp30, which enhanced antitumor capacity *in vitro* and *in vivo* (15). After exposure to neuroblastoma (NB) cells, NK-derived exosomes expressed higher levels of NCRs through unknown mechanisms; the exosomes were taken up by CD56^dim^ NK cells, which primed the naive cells to release more cytokines and upregulate NCR expression for more potent cytotoxicity against NB (17). However, it is unclear whether this effect is specific to certain tumor cell types. When NK cells encounter cytokines or a pathogen, they can develop stable, heritable properties known as immunologic memory, which may be in the form of surface molecules or exosome cargo and may enhance the cytotoxic effects of naive NK cells. Thus, NKEVs can potentially be exploited to improve host defense.

### Tumor-Homing Ability of NKEVs

EVs are mainly distributed in the spleen and liver where EVs are metabolized; the biodistribution is affected by dose, route of injection, and cellular origin of EVs (26). Several studies have reported the tumor-targeting ability of NKEVs (15, 27, 28). In a mouse glioblastoma xenograft model, a fluorescent signal was observed in tumors as early as 12 h after intravenous injection of NK-92 cell-derived exosomes, with the peak signal at 48 h (15). In another NB tumor-bearing mouse model, NK-92 cell-derived exosomes used as a drug delivery system showed good targeting ability, with strong fluorescence observed 6 h after injection; in mice with subcutaneous tumors, exosomes were detected in tumor tissues as early as 20 min after administration (27). Additionally, exosomes released by NK cells primed with IL-15 were faster-acting, had stronger targeting ability, and persisted for a longer period at the tumor site and in the circulation, with a half-life of up to24 h (15); the latter may be related to the expression of CD47, which serves as a deterrent signal to macrophages to evade immune clearance (29). The targeting mechanism of NKEVs is not known, but it was suggested that NCRs on the vesicle surface such as TRAIL, NKp30, and NKp44 or adhesion molecules such as lymphocyte function-associated antigen (LFA)-1/intercellular adhesion molecule (ICAM)-1 are involved in tumor cell recognition and targeting (15, 30). Additionally, the interaction of C-X-C chemokine receptor type (CXCR)4 (a chemokine receptor that mediates leukocyte trafficking) with stromal cell-derived factor (SDF)-1 (which plays an important role in cancer metastasis (31) and is expressed on exosomes released by ex vivo-expanded NK cells) was suggested to underlie the tumor-homing ability of vesicles in mice with CHLA-255 NB cell-derived tumors (27). NKEVs can also be taken up by normal cells and show immunomodulatory effects on immune cells, which can lead to side effects following systematic administration. However, in most studies NKEVs did not show significant cytotoxicity in normal cells even after 48 h of exposure.

### Cellular Uptake of NKEVs

NKEVs are known to be internalized by tumor cells including breast cancer (MCF-7 and MDA-MB-231/F) (9, 15, 16), acute lymphoblastic leukemia (Jurkat and NALM-18) (6, 16), melanoma (B16F10) (8), NB (D54F) (11), and anaplastic thyroid cancer (CAL-62/F) (15) cells as well as by normal cells such as astrocytes (19) and NK cells (17). The actual time required for uptake depends on the exosome and target cell; the minimum time reported to date is 30 min by NALM-18 childhood B acute lymphoblastic leukemia cells (14). NKEVs may transfer cargo to recipient cells *via* plasma membrane fusion, clathrin-mediated endocytosis, or receptor-mediated internalization (12, 32). The cytotoxic effects of NKEVs were shown to be partly abolished by antibodies against Fas or Fas ligand (FasL), suggesting that ligand–receptor interaction is another mechanism by which NKEVs are targeted to tumor cells (6, 8), although this has yet to be demonstrated experimentally.

## NKEVs Exert Antitumor Effects *via* Cargo Transfer

### NKEVs Bear Proteins That Are Cytotoxic to Tumor Cells

**Figure 1:**
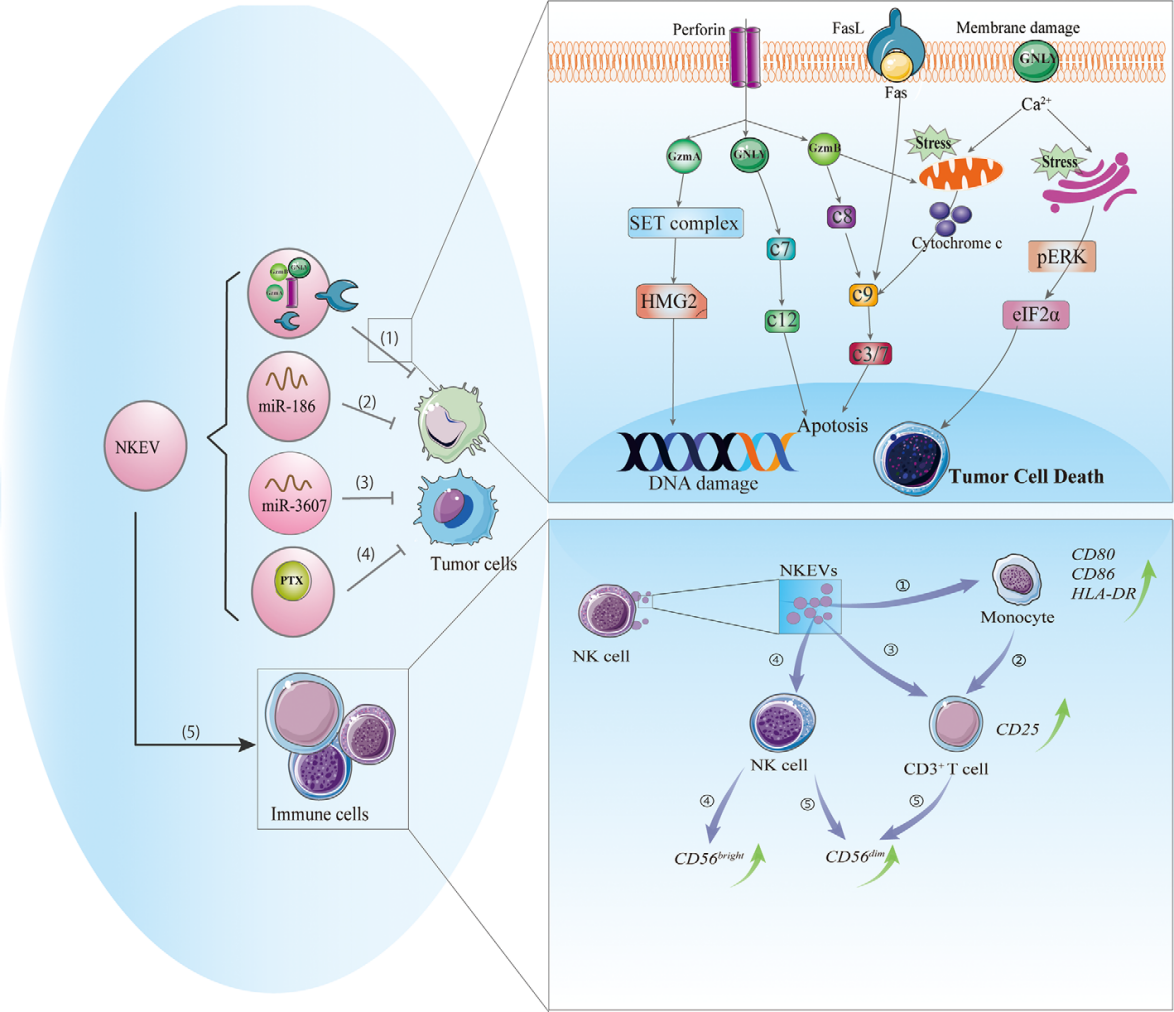
Cytotoxic and immunomodulatory effects of NKEVs. NKEVs carry numerous bioactive molecules such as cytotoxic proteins, microRNAs, and therapeutic drugs that can exert antitumor effects. (1) NKEVs containing cytotoxic proteins can kill tumor cells through caspase-dependent and -independent apoptotic pathways. Granzyme A induces the cleavage of the SET protein complex, leading to single-stranded DNA damage. Granzyme B directly activates procaspase and disrupts mitochondria, causing the release of cytochrome c and activation of the apoptotic cascade. Granulysin damages the cell membrane and induces ER stress-mediated apoptosis. (2) MiR-186 in NKEVs downregulates MYCN and AURKA expression, thereby inhibiting cell proliferation and inducing apoptosis of neuroblastoma cells. (3) MiR-3607-3p in NKEVs inhibits pancreatic cancer cell migration and invasion by targeting IL-26. (4) NKEVs loaded with paclitaxel can kill breast cancer cells. (5) NKEVs can stimulate immune cells. 1) NKEVs induce the expression of costimulatory molecules on monocytes. 2) NKEVs activate T cells by acting on monocytes. 3) NKEVs directly activate T cells by inducing the upregulation of CD25. 4) NKEVs stimulate NK cells, predominately the CD56^bright^ subgroup. 5) In the presence of T cell and monocyte stimuli, NKEVs increase the percentage of CD56^dim^ NK cells. Abbreviations: AURKA, Aurora kinase A; GNLY, granulysin; GzmA, granzyme A; GzmB, granzyme B; c3/7, caspase3/7; c7, caspase 7; c8, caspase8; c9, caspase9; c12, caspase 12; IL-26, interleukin 26; MYCN, V-Myc avian myelocytomatosis viral oncogene neuroblastoma-derived; NKEV, NK cell-derived extracellular vesicle.

NK-92 cells and NK cells isolated from human PBMCs or mouse spleen can release exosomes or MVs containing cytotoxic proteins. The type, amount, and functions of these proteins can vary depending on the cell source, physiologic status, or pretreatment. There is no single protein that is responsible for the cytotoxic effects of NKEVs; perforin, granzyme A and B, and granulysin may all contribute (21). The possible cytotoxic mechanisms of NKEVs are discussed below (**Figure 1**).

#### Caspase-Dependent/-Independent Apoptosis

Perforin is a pore-forming protein that can directly insert into the target cell membrane (33). Similarly, perforin in NKEVs may allow granzyme in vesicles to enter the target cell and may also form a pore in the endosome, leading to its rupture and granzyme release (34). The entry of NKEVs into target cells can trigger caspase-dependent and -independent apoptosis. In the former, granzyme B directly activates procaspase, activating a signaling cascade that disrupts mitochondria and causes the release of cytochrome c, which activates caspase-9, -3, and -7 to induce apoptosis (16, 35). In caspase-independent cell death, granzyme A induces mitochondrial stress, leading to the release of reactive oxygen species that cause single-stranded DNA damage; it also targets nuclear proteins for degradation, leaving DNA vulnerable to the activity of nucleases and caspases (36). Meanwhile, granulysin directly damages the target cell membrane and induces endoplasmic reticulum (ER) stress-mediated apoptosis (37). Increased degradation of 3-hydroxy-3-methylglutaryl-coenzyme A reductase (HMG)2 and SET nuclear proto-oncogene (SET), cytochrome C release, and activation of caspase-3, -7, and -9 were observed in NKEV-treated tumor cells; the altered expression of ER-associated markers such as protein kinase R-like ER kinase (PERK) and phosphorylated eukaryotic initiation factor (eIF)2α suggested that ER stress was involved in these processes (21). Thus, caspase signaling and mitochondrial and ER stress play important roles in NKEV-induced cytotoxicity.

#### Fas/FasL Pathway

FasL is a type II transmembrane protein of the tumor necrosis superfamily of death factors (38) that can trigger the exogenous apoptotic pathway through activation of caspase-8 and -3 and poly(ADP-ribose) polymerase (PARP). FasL can be packed into EVs by a variety of cell types including NK cells (21, 39–41). The soluble and/or membrane-bound forms of FasL have been detected in most NKEVs (6, 11, 15) and are thought to act *via* distinct mechanisms including classic receptor–ligand interaction involving the membrane receptor FasL expressed on NK92 cell-derived exosomes, which exhibits time- and dose-dependent cytotoxic effects on melanoma cells (8); and the endocytic pathway, whereby NKEVs containing soluble FasL is taken up by target cells and interacts with intracellular structures to induce FasL-mediated cell death (6). However, the role of FasL in NKEV-induced cytotoxicity remains controversial, with most of the data coming from anti-Fas antibody blockade experiments. For example, in two studies using exosomes derived from NK-92 cells (one with IL-15 priming), the exosomes showed cytotoxic effects on melanoma (B16F10) and breast cancer (MDA-MB-231/F) cells that were abolished by anti-Fas antibody (8, 15). In contrast, several studies have demonstrated that although ex vivo-expanded NK cell-derived exosomes contain FasL, they may not be related to the cytotoxicity of NKEVs (6, 21). It is possible that the Fas/FasL pathway is influenced by both FasL expression on NKEVs and Fas expression on target cells.

### Micro RNA-Containing NKEVs Inhibit Tumor Cells

MicroRNAs ((miRNA), the most common type of extracellular RNA, are delivered by immune cell-derived vesicles in the tumor microenvironment (e.g., miR-155 and miR-21) and exert a wide range of downstream regulatory effects (42, 43). Although studies on miRNAs in NKEVs are scarce, there have been some notable findings. Ex vivo-expanded NK cell exosomes carrying miR-186 were cytotoxic to MYCN-amplified NB cells (13). MYCN is an oncogene commonly mutated in gliomas and is a member of the hard-to-target MYC family (44). Aurora kinase (AURK)A improves the stability of MYCN by inhibiting its protease-dependent degradation and is an alternative target in MYCN-directed therapies (44); miR-186 in NKEVs was shown to reduce MYCN and AURKA expression, thereby inhibiting proliferation and inducing apoptosis in NB cells. Exosomes bearing miR-186 were also taken up by NK cells, leading to downregulation of TGF-β receptor (TGFBR)1/2 and reversing the cytotoxic effect of TGF-β on NK cells (13). These results indicate that miR-containing NKEVs can exert antitumor effects against NB *via* multiple mechanisms. In pancreatic cancer cells (Mia PaCa-2 and PANC-1), miR-3607-3p encapsulated in NKEVs suppressed cell migration and invasion, while a decrease in miR-3607-3p level was associated with poor prognosis and tumor metastasis (18). Moreover, miR-3607-3p in NKEVs reduced the mRNA and protein levels of IL-26, which is known to play a role in cancer cell proliferation and metastasis and is highly expressed in pancreatic cancer tissues. However, whether the inhibitory effects of NKEVs on pancreatic cancer cells depend on the inhibition of IL-26 or other mechanisms remains to be determined. Nonetheless, the existing evidence suggests that NK cells can exert antitumor effects through exosome-mediated delivery of nucleic acids.

### NKEVs Transport Antitumor Drugs

Given their tumor-targeting capacity, NKEVs can be used as a vehicle to transport antitumor drugs or therapeutic molecules. For example, paclitaxel (PTX), a drug used to treat several types of cancer, can be encapsulated in NKEVs *via* electroporation; PTX-NKEVs showed a strong inhibitory effect on human breast cancer cells compared to the same dose of free PTX (9). However, it is unclear whether this effect was due to increased cellular concentrations of PTX or to the antitumor effect of NKEVs. The capacity of the NKEVs as a drug delivery system was evaluated more visually in another study. In a mouse model of NB, NKEVs loaded with the therapeutic miRNA let-7a was found to accumulate in tumor tissue (27) and inhibited cancer cell proliferation by targeting cell cycle regulators (45); a 2-photon fluorescence imaging study using an IVIS Spectrum *in vivo* imaging system demonstrated that the NKEVs were efficiently targeted to the cancer site and delivered a high dose of let-7a (27). These results suggest that NKEVs can be used for targeted delivery of therapeutic molecules to tumor sites to induce tumor cell apoptosis.

NKEVs have also been shown to inhibit tumor cell proliferation although the underlying mechanism is not known. Mesenchymal stem cell-derived EVs were reported to inhibit tumor growth *via* modulation of mitogen-activated protein kinase (MAPK) signaling (46). The MAPK and protein kinase B (AKT)/phosphatidylinositol 3-kinase (PI3K) pathways were also found to be targeted by NKEVs, but their specific roles are unknown. Although one study has shown that TNF-α released by NKEVs affect tumor cell proliferation, survival, and apoptosis (8), the study did not address the mechanisms of action and the results indicated that NKEVs can exert antitumor effects in multiple ways.

## Immunomodulatory Effects of NKEVs

In addition to their cytotoxic activities, NK cells exert immunomodulatory effects by releasing EVs containing molecules that target the immune system *via* paracrine action or through the circulatory system (47). NKEVs can stimulate immune cells (**Figure 1**): T cell activation was increased in PBMCs cocultured with exosomes released by activated NK cells, as evidenced by the upregulation of CD25 in the CD3^+^ subset and enhanced release of cytokines, which was not observed in CD4+ T cells (7). Monocyte depletion experiments demonstrated that NKEVs can activate T cells either directly or indirectly by increasing the expression of costimulatory molecules on monocytes, thereby inducing T cell proliferation (7). NK cell-derived exosomes promoted M1 over M2 polarization in macrophages and increased the expression of inducible nitric oxide synthase (iNOS) while inhibiting that of arginase (ARG)-1 (48). NKEVs were also shown to stimulate NK cells, predominately those of the CD56^bright^ phenotype; notably, the presence of T cells and monocytes increased the fraction of CD56^dim^ NK cells. Furthermore, naive NK cells exposed to exosomes derived from NK cells preexposed to NB cells showed elevated expression of NCRs and had higher cytotoxicity against NB cells (17).

NKEVs may partly alleviate the immunosuppressive effects of tumor cells. TGF-β can inhibit the recognition and clearance of tumor cells by immune cells (49–51) and is used along with IL-10 or lipopolysaccharide to mimic an immunosuppressive environment. However, exposure to TGF-β did not diminish the antitumor capacity of NKEVs (13). Moreover, exosomes derived from ex vivo-expanded NK cells were capable of stimulating monocytes, T cells, and NK cells even under immunosuppressive conditions (7). Some studies have suggested that NKEVs harbor specific substances that act on the TGF-β pathway and thus alleviate immunosuppression (13, 52, 53). NKEVs were found to reduce the expression of programmed death (PD)-1—a major immune checkpoint molecule that is expressed by a variety of immune cells and plays an important role in immune escape by tumors (54)—on CD3/CD28-stimulated T cells (7). Additionally, NKEVs contain many molecules related to the immune response including IFN-γ, TNF-α, IL-10, MHC-I, and MHC-2, although their effects have not been well studied (10, 12, 14).

Immunosuppression is a mechanism by which tumor cells evade the immune response through continuous secretion of soluble factors and EVs or by inducing the expression of immune checkpoint molecules in immune cells. Enhancing antitumor immunity is a key strategy for cancer therapy; NKEVs can potentially modulate the immune response or even reverse tumor immunosuppression, making them a candidate agent for cancer immunotherapy.

## Conclusion and Future Perspectives

EVs are a promising alternative to cell-based therapies because of their nanoscale size, superior tissue penetration, and lower immunogenicity. NKEVs also have the ability to kill tumor cells and reactivate immune cells even under immunosuppressive conditions, which can help to overcome immune tolerance in tumors. Additionally, NKEVs can cross physiologic barriers, have tumor-targeting capacity, and resist immune clearance, making them highly attractive candidates for cancer therapy. However, large-scale production and storage, biodistribution, and heterogeneity are outstanding challenges that must be overcome for their clinical application.

Although NKEVs demonstrate good tumor targeting in mouse models, they can also accumulate in normal organs such as the spleen, liver, and kidney (15, 19). Although it is promising that in most studies NKEVs did not show significant cytotoxicity to normal cells even after 48 h of exposure (12, 14, 15), NKEVs are known to be taken up by normal cells and have shown cytotoxic effects in activated PBMCs (6), suggesting that they could have systemic side effects. Therefore, it is imperative to improve the targeting ability of NKEVs. Cytokine pretreatment is a potential strategy for achieving efficient and precise delivery; IL-15–pretreated NK cell-derived EVs showed faster action, enhanced tumor targeting, and a longer half-life compared to NKEVs without this priming (15). Additionally, source cells or exosomes can be engineered to express specific ligands targeting tumor tissues such as RVG peptide (targeting the central nervous system) (55), GE11 peptide (targeting epidermal growth factor receptor [EGFR]-expressing tumors) (56), and T7 peptide (targeting the cytosolic transferrin receptor [TfR] in glioblastoma) (57). NKEV targeting can also be improved using drugs: dextran sulfate inhibited exosome uptake by monocytes/macrophages by blocking scavenger receptor class A family (SR-A), thereby significantly reducing hepatic clearance of EVs in mice (58).

Another major barrier to the use of NKEVs for therapeutic purposes is their heterogeneity. In experimental settings, this can lead to variability across studies and reduce the reproducibility of the results. In clinical applications, this could present difficulties for bulk production of standard, uniform exosomes, which can cause problems for quality control, determination of therapeutic dose, and efficacy assessment. More detailed exploration of the biogenesis of EVs—e.g., through vesicle subpopulation analyses—can enable the isolation of specific types of vesicle and thus reduce heterogeneity at the source. In addition to EVs, NK cells are highly variable in terms of molecular profile and function. Few studies have addressed the diversity of EVs released by different NK cell subtypes or under different conditions, and the phenotypes and characteristics of source cells and their EVs have not been investigated in detail. It is well known that the CD56^dim^ NK cells exert cytotoxic effects whereas the CD56^bright^ subtype plays an important role in immunomodulation; however, it is unclear whether the EVs released by these two populations have distinct properties. A detailed characterization of EVs released by different NK cell subtypes can answer this question.

In conclusion, NKEVs have unique properties and antitumor activity that make them promising candidate agents for cancer immunotherapy. High-throughput molecular profiling as well as *in vivo* studies can advance our understanding of NKEVs and provide a basis for their development as therapeutic tools.

## Author Contributions

FW: Data curation and manuscript drafting. MX, MH, ZS, and CL: Reviewing and Editing. SL, XC, and WW: Preparing the figure and table. JT and CW: Conceptualization and revising. All authors contributed to the article and approved the submitted version.

## Conflict of Interest

The authors declare that the research was conducted in the absence of any commercial or financial relationships that could be construed as a potential conflict of interest.
